# Within-guild dietary discrimination from 3-D textural analysis of tooth microwear in insectivorous mammals

**DOI:** 10.1111/jzo.12068

**Published:** 2013-08-27

**Authors:** M A Purnell, N Crumpton, P G Gill, G Jones, E J Rayfield

**Affiliations:** 1 Department of Geology, University of LeicesterLeicester, UK; 2 School of Earth Sciences, University of BristolBristol, UK; 3 Department of Zoology, University of CambridgeCambridge, UK; 4 School of Biological Sciences, University of BristolBristol, UK

**Keywords:** insectivore microwear, carnivore, bats, dietary analysis, *Rhinolophus*, *Pipistrellus*, *Plecotus*, ISO roughness

## Abstract

Resource exploitation and competition for food are important selective pressures in animal evolution. A number of recent investigations have focused on linkages between diversification, trophic morphology and diet in bats, partly because their roosting habits mean that for many bat species diet can be quantified relatively easily through faecal analysis. Dietary analysis in mammals is otherwise invasive, complicated, time consuming and expensive. Here we present evidence from insectivorous bats that analysis of three-dimensional (3-D) textures of tooth microwear using International Organization for Standardization (ISO) roughness parameters derived from sub-micron surface data provides an additional, powerful tool for investigation of trophic resource exploitation in mammals. Our approach, like scale-sensitive fractal analysis, offers considerable advantages over two-dimensional (2-D) methods of microwear analysis, including improvements in robustness, repeatability and comparability of studies. Our results constitute the first analysis of microwear textures in carnivorous mammals based on ISO roughness parameters. They demonstrate that the method is capable of dietary discrimination, even between cryptic species with subtly different diets within trophic guilds, and even when sample sizes are small. We find significant differences in microwear textures between insectivore species whose diet contains different proportions of ‘hard’ prey (such as beetles) and ‘soft’ prey (such as moths), and multivariate analyses are able to distinguish between species with different diets based solely on their tooth microwear textures. Our results show that, compared with previous 2-D analyses of microwear in bats, ISO roughness parameters provide a much more sophisticated characterization of the nature of microwear surfaces and can yield more robust and subtle dietary discrimination. ISO-based textural analysis of tooth microwear thus has a useful role to play, complementing existing approaches, in trophic analysis of mammals, both extant and extinct.

## Introduction

Dietary analysis of mammals is central to a wide range of evolutionary, ecological and conservation issues. Resource exploitation and competition for food are important selective pressures in animal evolution, and understanding the linkages between diversification, trophic morphology and diet are critical to testing hypotheses of adaptive radiation and the roles of dietary niche partitioning and competition in speciation (e.g. Darwin, 1859; Schluter, 2000; Dayan & Simberloff, 2005; Price *et al*., 2012). A number of such investigations in recent years have focused on bats (Freeman, 2000; Nogueira, Peracchi & Monteiro, 2009; Dumont *et al*., 2012; Santana, Grosse & Dumont, 2012). Among mammals, bats are ideally suited for such analyses because their roosting habits and the accessibility of roosts mean that for many species diet can be quantified relatively easily through faecal analysis (Kunz & Whitaker, 1983). Although such ‘scat analysis’ is becoming more widespread (Trites & Joy, 2005; Williams, Goodenough & Stafford, 2012), in some cases supplemented by DNA-based identification of prey species (Razgour *et al*., 2011), dietary analysis in mammals is otherwise invasive, complicated, and time consuming. It is therefore expensive, requiring detailed analysis of stomach or cheek-pouch contents, the contents of food stores or direct behavioural observation (e.g. Jordan, 2005). Analysis of stable isotopes of C and N can also be informative, especially in marine mammals (e.g. Kelly, 2000), but this usually provides only an indication of relative trophic levels.

Analysis of the patterns of wear on teeth that arise as a consequence of feeding provides an alternative route to dietary discrimination, with an established track record of application to mammals (e.g. Walker, Hoeck & Perez, 1978; Gordon, 1982; Teaford, 1988). In particular, analysis of microwear – the microscopic chipping and scratching within wear facets – can provide insights into the jaw kinematics and trophic ecology of species where other data are unavailable. It can be applied to historical museum specimens and extinct taxa for example. Furthermore, because the dietary signal of microwear accumulates over periods of days or weeks (Teaford & Oyen, 1989; Merceron *et al*., 2010) analysis of microwear avoids the problem of stomach contents recording only the ‘snapshot’ of what an animal ate in the few hours prior to capture (Merceron *et al*., 2010; Purnell, Seehausen & Galis, 2012). Microwear analysis has a long history of application to primates and ungulates in particular (e.g. Walker *et al*., 1978; Teaford, 1988; Scott *et al*., 2005), but new approaches to examination and quantification of wear patterns are allowing microwear analysis to be applied to new problems and to a broader range of taxa, including carnivorans, dinosaurs and fish (Scott *et al*., 2005, 2006; Purnell *et al*., 2006, 2007, 2012; Ungar, Merceron & Scott, 2007; Ungar, Grine & Teaford, 2008; Goillot, Blondel & Peigne, 2009; Williams, Barrett & Purnell, 2009; Merceron *et al*., 2010; Schubert, Ungar & DeSantis, 2010; Schulz, Calandra & Kaiser, 2013*a* 2013a; Schulz *et al*., 2013*b* 2013b).

Here we present evidence from insectivorous bats that analysis of three-dimensional (3-D) textures of tooth microwear using ISO roughness parameters derived from sub-micron surface elevation data (International Organization for Standardization, 2012) provides an additional, powerful tool for investigation of trophic resource exploitation in mammals. Our approach is based on the same type of high-resolution 3-D data and offers the same advantages as scale-sensitive fractal analysis (SSFA) of tooth microwear (Scott *et al*., 2005, 2006; Ungar *et al*., 2007, 2008; Merceron *et al*., 2010). These advantages include improvements in robustness, repeatability and comparability of studies, realized to a large extent because 3-D approaches are not dependent on operators to identify, measure and score scratches and pits on tooth surfaces, a problem that creates significant noise and error in two-dimensional (2-D) microwear analyses (Grine, Ungar & Teaford, 2002; Purnell *et al*., 2006; Mihlbachler *et al*., 2012).

Only a few studies have investigated microwear in carnivorous mammals (Taylor & Hannam, 1986; Van Valkenburgh, Teaford & Walker, 1990; Strait, 1993*a* 1993a; Anyonge, 1996; Goillot *et al*., 2009; Schubert *et al*., 2010; Bastl, Semprebon & Nagel, 2012; DeSantis *et al*., 2012). Of these, all except for Strait (1993*a*) are studies of large carnivores, and only two utilize the analytically more robust 3-D approaches (Schubert *et al*., 2010; DeSantis *et al*., 2012). These studies demonstrate the potential for analysis of microwear to discriminate between carnivores with different diets, but Strait’s (1993*a*) work remains the only previous analysis of microwear in bats. Using scanning electron microscopy (SEM) to quantify microwear in small-bodied bats and primates, Strait was unable to distinguish insectivores from flesh eaters, but found significant differences between species that consumed hard and soft prey. The hypothesis that microwear differs within a guild of small-bodied insectivores remains untested. This study, which explores this hypothesis, also provides the first application of 3-D textural analysis to small-bodied mammals.

## Materials and methods

Insectivores with well-constrained differences in their diets were selected for this analysis. We analysed four species of bat: common pipistrelle *Pipistrellus pipistrellus*, soprano pipistrelle *Pi. pygmaeus*, brown long-eared bat *Plecotus auritus* and greater horseshoe bat *Rhinolophus ferrumequinum*. Specimens were all wild-found, and acquired from UK sources (see Supporting Information).

In considering the dietary differences between insectivorous animals, what matters is not the taxonomic identity of the prey, but the relative difficulty faced by the predator when attempting to pierce and chew the prey items. Terminology used to characterize the relevant properties of prey items is complicated (for discussion see Evans & Sanson, 2005; Freeman & Lemen, 2007). Evans & Sanson (2005) suggested the term ‘intractability’, but this is not widely used and can be confusing. Here we use ‘hard’ and ‘soft’ to mean prey that is more or less difficult to pierce and chew.

Information regarding diets of the bat species studied comes mainly from Barlow (1997) and Vaughan (1997) and references therein. *Pipistrellus pipistrellus* and *Pi. pygmaeus* were only recently recognized to be separate, cryptic species based on molecular, behavioural and echolocation differences (Jones & Van Parijs, 1993; Barratt *et al*., 1997); their diets are subtly different. Both are specialists on Diptera (flies) with a preference for Nematocera (mosquitoes, crane flies, gnats, and midges), but they consume different families in different proportions. *Pipistrellus pipistrellus* consumes more non-nematoceran dipterans and other insects with a wider range of cuticle ‘hardness’ in its diet [greater quantities of Trichoptera (caddisflies), Neuroptera (lacewings), Hymenoptera (sawflies, wasps, bees and ants), Lepidoptera (moths and butterflies) and Coleoptera (beetles)] (Swift, Racey & Avery, 1985; Barlow, 1997). This diet includes ‘harder’ prey than that of *Pi. pygmaeus*, the diet of which is made up mostly (*c.* 80%) of the ‘softer’ ‘biting’ and ‘non-biting’ midges (Table 1; Barlow, 1997). Also, *Pi. pipistrellus* is known to consume larger flies than *Pi. pygmaeus*, and the ‘hardness’ of insects is correlated with size (Aguirre *et al*., 2003; Freeman & Lemen, 2007). In summary, *Pi. pipistrellus* consumes prey that spans a broad range of ‘hardness’, whereas the prey of *Pi. pygmaeus* is narrower in range and generally ‘softer’.

**Table 1:** Trophic categorization and diets of the British bat species analysed, modified from Vaughan (1997 and references therein) and Barlow (1997)

Species	Trophic category	Diet
Common pipistrelle *Pipistrellus pipistrellus*	Prey of mixed ‘hardness’; Diptera specialist, but with some ‘harder’ species	Mostly suborder Nematocera: Psychodidae ‘moth flies’; Anisopodidae ‘wood gnats’; Muscidae ‘house flies’.
Soprano pipistrelle *Pipistrellus pygmaeus*	Diptera specialist, particularly midges; mainly ‘softer’ prey	Mostly suborder Nematocera: Chironomidae ‘non-biting midges’; Ceratopogonidae ‘biting midges’.
Greater horseshoe bat *Rhinolophus ferrumequinum*	Prey of mixed ‘hardness’; mixed feeder, including more ‘hard’ prey, especially Coleoptera	Mainly Lepidoptera & Coleoptera. Lepidopteran families: Noctuidae ‘owlet moths’; Nymphalidae ‘brush-footed butterflies’; Hepialidae ‘swift moths’; Sphingidae ‘hawk moths’; Geometridae ‘geometer moths’; Lasiocampidae ‘lappet moths’. Coleopteran families: Scarabaeidae ‘scarab beetles’; Geotrupidae ‘dor beetles’; Silphidae ‘carrion beetles’; Carabidae ‘ground beetles’. Diptera also consumed.
Brown long-eared bat *Plecotus auritus*	‘Soft’ prey specialist; specializing on Lepidoptera	Almost entirely Lepidoptera: Noctuidae ‘owlet moths’; Hepialidae ‘swift moths’; Thyatiridae Nymphalidae ‘brush-footed butterflies’; Geometridae ‘geometer moths’; Sphingidae ‘hawk moths’; Notodontidae ‘prominents’; Arctiidae Pyralidae ‘snout moths’.

Assessment of prey ‘hardness’ was based on published data (cited in text).

*Rhinolophus ferrumequinum* is a mixed forager, consuming Lepidoptera (butterflies and moths) and Coleoptera (beetles) in approximately equal amounts, together with dipterans (Jones, 1990). Like *Pi. pipistrellus*, this diet is a mixture of ‘soft’ prey, and prey that is among the ‘hardest’ of insects (i.e. coleopterans). *Plecotus auritus* specializes on Lepidoptera, with faecal studies indicating that Lepidoptera can constitute 99–100% of the diet: Lepidoptera are known from numerous studies to be among the ‘softest’ insects (Aguirre *et al*., 2003; Evans & Sanson, 2005; Freeman & Lemen, 2007).

Rather than extract individual teeth, mandibles were removed from entire cadavers (see Supporting Information for preparation details). For all specimens, data were acquired from the distal wear facet of the M_2_ protoconid – as near to the cusp tip as possible without compromising surface flatness – because of the significant role it plays in food processing (Strait, 1993*b*).

Our methods for data acquisition and analysis are modified slightly from those of Purnell *et al*. (2012). Before sputter coating with gold (SC650, Bio-Rad, Hercules, CA, USA), specimens were mounted onto 12.7 mm SEM stubs [using carbon disks and Leit-C plastic carbon cement (Fluka, Buchs, Switzerland)], with the M_2_ facet of interest oriented horizontally in order to maximize the quality of data acquired.

High-resolution 3-D surface data were captured using an Alicona Infinite Focus microscope G4b (IFM; Alicona GmbH, Graz, Austria; software version 2.1.2), using ×100 objective to give a field of view of 145 × 110 *μ*m. Recent work (Merceron *et al*., 2010; Purnell *et al*., 2012) has show that this is a large enough area to extract dietarily informative texture data; furthermore, many of the teeth analysed here are too small for a larger area to be sampled. The Alicona Infinite Focus microscope G4b has a CCD of 1624 × 1232 pixels. In theory, for a field of view of 145 *μ*m, this equates to a lateral sampling distance of 0.09 *μ*m, but the limits imposed by the wavelength of white light mean that lateral optical resolution is actually about 0.35–0.4 *μ*m. For all samples, vertical resolution was set at 20 nm, and the lateral resolution factor for the IFM was set at 0.3. Exposure and contrast settings were adjusted to maximize data quality in terms of measurement repeatability (this is estimated automatically by the IFM software during data capture) for each sample. Adjusting exposure and contrast do not affect the values for 3-D measurements. Prior to generation of roughness surfaces, captured 3-D surface data for each specimen was examined visually to ensure that only those surfaces which preserved primary tooth microwear textures were subject to analysis. Data showing evidence of post-mortem artefacts or with extraneous material obscuring the surface were rejected.

**Figure 1:**
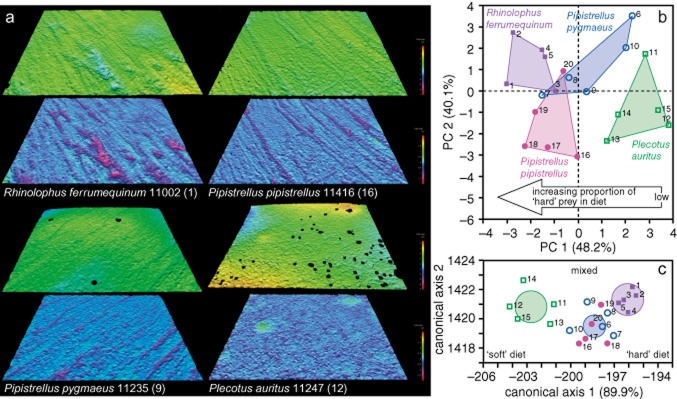
Tooth microwear textures of bats, and multivariate analysis of International Organization for Standardization (ISO) roughness parameters. (a) Digital elevation models showing levelled surface data (above) and scale-limited roughness surfaces (below) for the four species of bats. See text for details of data processing; numbers in brackets identify specimens in (b) and (c). Measured areas are 146-*μ*m wide. (b) Principal components (PC) analysis of ISO roughness parameters that differ between species. Species form largely non-overlapping clusters; PC axis 1 correlates with dietary differences between species. For details of loadings (eigenvectors) of roughness parameters onto PC axes 1 and 2 see Supporting Information Table S3. (c) Linear discriminant analysis of ISO roughness parameters that differ between species. Analysis correctly assigns all specimens to one of three trophic groups (groups based on the amount of ‘hard’ prey consumed; probability of correct assignment >0.9 for all but one *Pipistrellus pygmaeus* (0.64) and one *Pipistrellus pipistrellus* (0.63); Wilks’ Lambda = 0.07; *F* = 2.72; *P* = 0.02). Canonical axis 1 correlates with dietary differences between species. Ellipses show 95% confidence limits for means.

All 3-D data were processed using the Alicona IFM software (version 2.1.2) to remove dirt and dust particles from the surface (by manual deletion), and were then exported as .sur files for processing using SurfStand software (version 5.0.0 Centre for Precision Technologies, University of Huddersfield, West Yorkshire, UK). Measurement errors (anomalous peaks and low points) were deleted, and data were levelled (subtraction of least squares plane) to remove variation caused by differences in orientation of tooth surfaces at the time of data capture. Scale-limited roughness surfaces were generated from the data through application of a fifth-order robust polynomial (which finds and removes the least squares fifth-order polynomial surface for the levelled data) and a robust Gaussian wavelength filter (*λ*_c_ = 0.025 mm; to remove long wavelength features of the tooth surface (gross tooth form; Fig. 1). ISO 25178-2 texture parameters (International Organization for Standardization, 2012) were then generated from the resulting roughness surface. These include: height parameters (quantifying the distribution of height values along the *z*-axis); spatial parameters (quantifying direction and spatial periodicity of the surface); hybrid parameters (combining the information present on the *x*-, *y*-and *z*-axes of the surface, quantifying aspects of the spatial shape of the data), and parameters related to measures of volumes, such as peak material, calculated from the areal material ratio curve (see Supporting Information). Sample sizes used in this study are relatively small (five individuals of each species), but as demonstrated by Purnell *et al*. (2012), this does not prevent detection of dietary signals through textural analysis of microwear.

Data were explored using analysis of variance (ANOVA), correlations, principal components analysis (on correlations; PCA) and linear discriminant analyses (LDA). All statistical analysis of microtextural data was carried out using JMP 9 (SAS Institute, Cary, NC, USA). The results of Shapiro–Wilk tests indicated that some roughness parameters were non-normally distributed (*P* > 0.05), and log-transformed data were used for analysis (the only exception was Ssk, which included negative values and for which we could not reject the null hypothesis that data were drawn from a population with a normal distribution). Where homogeneity of variance tests (Bartlett and Levene tests) revealed evidence of unequal variances, Welch ANOVA was used. The significance of LDA was assessed using Wilks’ Lambda.

## Results

ANOVA revealed that nine parameters differed significantly between bat species (Table 2 ). The nine parameters are: Ssk – skewness of the surface; Str – texture aspect ratio; Vmp – peak material volume; Vmc – core material volume; Vvc – core void volume; Vvv – dale void volume; Svk – reduced dale height; Smr1 and Smr2 – material ratio for peaks and dales, respectively. Core, peaks and dales are defined by the bearing area curve for the scale limited surface; for more detailed description and discussion of parameters see ISO 25178-2 (International Organization for Standardization, 2012) and Supporting Information. Tukey’s honestly significant difference (HSD) procedure indicates that *R. ferrumequinum* differs significantly from *Pl. auritus* for six of the nine parameters (lower Ssk, and Str, higher Vmc, Vvc, Vvv and Svk); it differs from *Pi. pipistrellus* for three (Vmc, Vvc, Vvv; all higher in *R. ferrumequinum*), but does not differ from *Pi. pygmaeus*. *Plecotus auritus* differs from *Pi. pipistrellus*, for four parameters (Ssk, Str, Smr1 and Smr2; all higher in *Pl. auritus*), and from *Pi. pygmaeus* for Vvc and Vvv (lower in *Pl. auritus*). The two *Pipistrellus* species differ only for Vmp and Vvc (higher in *Pi. pygmaeus*).

**Table 2:** Results of analysis of ANOVA, bat roughness parameters (log transformed)

	d.f.	*F*	*P*
Sq	3, 16	1.715	0.204
**Ssk**	**3, 16**	**6.733**	**0.004**
Suk	3, 16	0.078	0.971
Sp	3, 16	0.457	0.716
Sv	3, 16	1.091	0.381
Sz	3, 16	0.358	0.784
Sds	3, 16	0.468	0.709
**Str**	**3, 16**	**10.020**	**0.0006**
Sal	3, 16	2.923	0.066
Sdq^a^	3, 8.24	2.231	0.160
Ssc^a^	3, 7.54	0.833	0.514
Sdr^a^	3, 8.23	1.688	0.244
**Vmp**	**3, 16**	**3.364**	**0.045**
**Vmc**	**3, 16**	**10.413**	**0.0005**
**Vvc**	**3, 16**	**7.988**	**0.002**
**Vvv**	**3, 16**	**16.697**	**<.0001**
Spk^a^	3, 8.47	2.221	0.159
Sk	3, 16	1.763	0.195
**Svk**	**3, 16**	**5.508**	**0.009**
**Smr1**^a^	**3, 8.65**	**7.138**	**0.010**
**Smr2**	**3, 16**	**5.704**	**0.007**
S5z	3, 16	0.163	0.920
Sa	3, 16	2.140	0.135

Parameters in bold are those for which the null hypothesis of no difference between species can be rejected. ^a^Indicates Welch test result (ANOVA, unequal variances, Bartlett and/or Levene test). ANOVA, analysis of variance; d.f., degrees of freedom.

PCA of these nine parameters (Fig. 1) reveals that bat species are separated according to dietary preferences in a space defined by PC axes 1 and 2. PC axis 1 (48.2% of variance) is strongly correlated with bats dietary preferences (*r*_s_ = 0.81, *P* < 0.0001; bats ranked according to proportion of ‘hard’ prey in diet: *R. ferrumequinum* 1, *Pi. pipistrellus* 2, *Pi. pygmaeus* 3, *Pl. auritus* 4). The ‘soft’ diet specialist (*Pl. auritus*) has positive values, while *R. ferrumequinum*, which consumes the highest amounts of ‘hard’ prey, has negative values. The two *Pipistrellus* species span the gap between, with *Pi. pipistrellus* overlapping with *R. ferrumequinum* on PC axis 1, while the range of values for *Pi. pygmaeus* extends to include some that are similar to *R. ferrumequinum* and some that are similar to *Pl. auritus*.

Analysis of bat surface texture parameters thus defines a ‘dietary space’ in which increasingly negative values for PC axis 1 indicate higher proportions of ‘hard’ prey, while increasing positive values indicate decreasing proportions of ‘hard’ prey. ANOVA of the PCA results provides further support: PC axes 1 and 2 both differ between species [PC axis 1, *F* = 14.97; degrees of freedom (d.f.) = 3, 13; *P* < 0.0001; PC axis 2, *F* = 4.97; d.f. = 3, 13; *P* = 0.013]. Tukey’s HSD procedure reveals that for PC axis 1 *R. ferrumequinum* differs from *Pl. auritus* and *Pi. pygmaeus*, *Pl. auritus* differs from *R. ferrumequinum* and *Pi. pipistrellus*. The two *Pipistrellus* species do not differ from one another. For PC axis 2, *Pi. pipistrellus* differs from *R. ferrumequinum* and *Pi. pygmaeus*. That the bat species are separated into largely non-overlapping areas of space defined by the first two axes of a PCA based solely on ISO roughness parameters derived from worn tooth surfaces, and that there are significant differences between species, provides powerful evidence that microtextural analysis of tooth microwear can differentiate between species within trophic guilds, in this case between insectivores, for some of which dietary differences are quite subtle.

LDA produced a similar result to PCA analysis (Fig 1). Bat species were assigned to three dietary groups based on the amount of ‘hard’ prey consumed: higher (*R. ferrumequinum*) mixed (*Pipistrellus* species) and lower (*Pl. auritus*). LDA based on the nine roughness parameters assigned 100% of bat specimens to their correct dietary group. As with PCA, canonical axis 1 (89.9% variance) is strongly correlated with diet (species ranked 1–4; *r*_s_ = −0.84, *P* < 0.0001).

Our results also allow us to explore the relationship between ISO roughness parameters and diet. Rank correlation of dietary preferences reveals that 10 parameters are correlated with diet (*P* < 0.05; Table 3). Ssk, Str, Smr1, Smr2 and Sa decrease as the proportion of ‘hard’ prey increases; these are parameters that capture aspects of the height (Ssk, Sa), spatial (Str), and areal material ratio attributes of the roughness surface (Smr1, Smr2). Sq, Vmc, Vvv, Sk and Svk increase with increasing proportion of ‘hard’ prey; these are parameters that capture aspects of the height (Sq) and areal material ratio attributes of the roughness surface (Sk, Svk, Vmc, Vvv; the latter two capturing core material and valley void volume). In simple terms, as the amount in ‘hard’ prey increases, tooth surfaces tend to have deeper valleys, the elevations of the surface and the core material (i.e. not peaks or valleys) are higher, there are fewer peaks, and there is more directionality to the surface texture. This is illustrated in Fig. 1a: *R. ferrumequinum* sample 11002 exhibits roughness values that, for seven of the 10 diet-correlated parameters, are towards the ‘hard’ end of the dietary scale; *Pl. auritus* sample 11247, on the other hand, exhibits values for seven of the parameters that are towards the ‘soft’ end.

**Table 3:** Correlations between dietary rank and ISO roughness parameters (*n* = 20)

Parameter	Spearman’s ρ	*P*
**Sq**	**−0.465**	**0.039**
**Ssk**	**0.714**	**0.000**
Sku	0.023	0.922
Sp	0.209	0.376
Sv	−0.124	0.602
Sz	0.016	0.948
Sds	0.116	0.625
**Str**	**0.683**	**0.001**
Sal	0.241	0.306
Sdq	−0.066	0.782
Ssc	0.023	0.922
Sdr	0.016	0.948
Vmp	−0.147	0.535
**Vmc**	**−0.621**	**0.004**
Vvc	−0.372	0.106
**Vvv**	**−0.706**	**0.001**
Spk	0.279	0.233
**Sk**	**−0.512**	**0.021**
**Svk**	**−0.706**	**0.001**
**Smr1**	**0.594**	**0.006**
**Smr2**	**0.621**	**0.004**
S5z	−0.132	0.580
**Sa**	**−0.528**	**0.017**

See Supporting Information for definitions of parameters. Significant correlations are shown in bold.

## Discussion

Our method of data acquisition (focus variation microscopy) differs from previous applications of 3-D textural analysis of tooth microwear to dietary discrimination in mammals (which used confocal microscopy or interferometry). Most previous analyses employed SSFA, but our analysis is based on ISO parameters generated from scale-limited roughness surfaces (c.f. Purnell *et al*., 2012; Schulz *et al*., 2013*a*). Nevertheless, our results provide further confirmation of the power of 3-D textural analysis of tooth microwear as a tool for dietary discrimination. The results of PCA analysis, which requires no prior assumptions regarding dietary preferences or tooth wear in the species under investigation, are particularly compelling: the four bat species occupy largely non-overlapping areas in the space defined by PC axes 1 and 2, with clear separation between the species that eats the most ‘hard’ prey (*R. ferrumequinum*) and the ‘soft’ prey specialist (*Pl. auritus*). This result, coupled with statistical testing and linear discriminate analysis, demonstrates clearly that the 3-D texture of microwear as captured by ISO roughness parameters carries a strong dietary signal, and can detect subtle dietary differences between even cryptic species of insectivore.

Only a few previous analyses (Calandra *et al*., 2012; Purnell *et al*., 2012; Schulz *et al*., 2013*a*,*b*) have used ISO roughness parameters to investigate dietary differences between extant animals. The parameters found by Purnell *et al*. (2012) to differ significantly between cichlid fishes are not the same as for bats, but in both studies volume parameters (Vmp, Vmc, Vvc and Vvv for cichlid lower pharyngeal jaws; Vmp, Vmc, Vvc for cichlid oral teeth) differ between animals with different diets. In terms of diet, comparing results for cichlid lower pharyngeal jaws with the bats, it is the individuals that consume the most ‘hard’ food items (mollusc shells in the cichlids; beetle cuticle in bats) that have the higher values for volume parameters. Comparing insectivorous bats with cichlids that scrape-up algae (cichlid oral teeth analysis of Purnell *et al*., 2012) is a little more difficult, but for both groups, higher values for volume parameters occur in the animals in which teeth encounter more hard materials (rock scraping *Neochromis gigas* in the cichlids).

The texture parameters found by Schulz *et al*. (2013*a*) to differ between grazing and browsing ungulates also include three of the volume parameters that differ between bats. Vmc, Vvc, and Vvv are higher in grazers, interpreted by Schulz *et al*. (2013a) to reflect their more abrasive diet. Calandra *et al*. (2012) investigated six ISO roughness parameters in primates, only two of which (Sq and Vm) are directly comparable with parameters calculated here for bats. They found fairly weak statistical support for differences in parameter values between taxa, but observed that large hard particles produce tooth surfaces with more microscopic relief, with the highest Vm values found in species with a high proportion (>50%) of fruit (and therefore seeds) in their diet. This is consistent with our evidence of higher values for volume parameters in bats, which consume the highest amounts of ‘hard’ prey. Because they found few significant differences in ISO parameters between primates with different diets, Calandra *et al*. (2012) concluded that SSFA of microwear is a better tool for dietary discrimination. However, our results and those of Schultz *et al*. (2013a) suggest that analysis based on ISO parameters has comparable discriminatory power.

Comparing our results to the early work by Strait (1993*a*), her 2-D SEM-based approach went some way to demonstrating the potential of microwear analysis for dietary discrimination in bats: she was able to detect differences between hard object and soft object feeders, but was unable to discriminate between insectivores and flesh eaters. ISO roughness parameters provide a much more sophisticated characterization of the nature of microwear surfaces than is possible with 2-D analysis. Our results demonstrate that ISO-based analysis, in addition to avoiding the problems inherent in microwear analysis based on operator scoring, is capable of more robust and more subtle dietary discrimination.

Further work is required, including more detailed comparisons of different approaches to data acquisition, processing and analysis, with more comparative evaluations of ISO and SSFA approaches, but our results demonstrate the potential of ISO-based textural analysis for dietary discrimination, and establish the first set of ISO roughness data from extant small-bodied insectivorous mammals with known diets. These data provide textural reference points that will allow future studies to use ISO roughness characterization of microwear to test hypotheses of dietary specialization, niche partitioning and validation of functional models for taxa where dietary data are otherwise difficult to obtain, including extinct early mammals, many of which are assumed to have been insectivores.

## Supporting Information

**Figure S1** The areal material ratio curve (also referred to as the bearing area curve, or Abbot–Firestone curve) from which a number of height, volume, and material ratio parameters are derived. For definitions of parameters, see Supporting Information Table S1. The curve is a cumulative probability density function, derived from the scale-limited surface by plotting the cumulative percentage of the surface against height. Core, peaks and valleys within a surface are defined on the basis of this curve, with the core equivalent to the volume that lies between the heights of the surface delimited by the extrapolated intercept of the minimum slope of the curve as shown in the figure. Modified with permission from Alicona Infinite Focus Manual. **Figure S2** Cross-section through a surface showing how volume parameters relate to a surface. Note that this is a two-dimensional profile, but the parameters are volumes calculated for the whole surface. Modified with permission from Alicona Infinite Focus Manual. **Table S1** Specimens from which microtextural data were acquired. During preparation, the M_3_, coronoid crest and all other morphology posterior to the M_2_ was removed on both sides of the mandible to allow unobstructed examination of the M_2_. Soft tissues were removed from the teeth and jaws, carefully avoiding any contact between instruments and the M_2_ crowns, and specimens were further cleaned by boiling in individual beakers of water for 2 min. Prior to analysis, tooth surfaces were carefully cleaned with acetone applied with a soft synthetic brush. In a few cases the first attempts at data acquisition from the M_2_ facets revealed the presence of small crystals precipitated over the functional surface. These specimens were returned for a short period to the solution in which they had been stored, were gently brushed with de-ionized water on removal, and air-dried before examination. **Table S2** Short definitions and categorization of three-dimensional areal surface texture parameters. For further explanation see Supporting Information Figs S1 and S2. **Table S3** Loadings (eigenvectors) for roughness parameters onto PC axes 1 and 2 for the principal components (PC) analysis (nine parameters that differ significantly between bat species).
