# Supplementary material for: Within-guild dietary discrimination from 3-D textural analysis of tooth microwear in insectivorous mammals
Source: J Zool (1987). 2013 Aug 27;291(4):249–57. doi: 10.1111/jzo.12068 (PMC4296236; doi:10.1111/jzo.12068)
Supplement: Supplementary file 1 — Figure S1 The areal material ratio curve (also referred to as the bearing area curve, or Abbot–Firestone curve) from which a number of height, volume, and material ratio parameters are derived. For definitions of parameters, see Supporting Information Table S1. The curve is a cumulative probability density function, derived from the scale-limited surface by plotting the cumulative percentage of the surface against height. Core, peaks and valleys within a surface are defined on the basis of this curve, with the core equivalent to the volume that lies between the heights of the surface delimited by the extrapolated intercept of the minimum slope of the curve as shown in the figure. Modified with permission from Alicona Infinite Focus Manual. Figure S2 Cross-section through a surface showing how volume parameters relate to a surface. Note that this is a two-dimensional profile, but the parameters are volumes calculated for the whole surface. Modified with permission from Alicona Infinite Focus Manual. Table S1 Specimens from which microtextural data were acquired. During preparation, the M3, coronoid crest and all other morphology posterior to the M2 was removed on both sides of the mandible to allow unobstructed examination of the M2. Soft tissues were removed from the teeth and jaws, carefully avoiding any contact between instruments and the M2 crowns, and specimens were further cleaned by boiling in individual beakers of water for 2 min. Prior to analysis, tooth surfaces were carefully cleaned with acetone applied with a soft synthetic brush. In a few cases the first attempts at data acquisition from the M2 facets revealed the presence of small crystals precipitated over the functional surface. These specimens were returned for a short period to the solution in which they had been stored, were gently brushed with de-ionized water on removal, and air-dried before examination. Table S2 Short definitions and categorization of three-dimensional areal surface texture par [file jzo0291-0249-sd1.docx]

**Supplementary information**

Table S1. Specimens from which microtextural data were acquired. During preparation, the M_3_, coronoid crest and all other morphology posterior to the M_2_ was removed on both sides of the mandible to allow unobstructed examination of the M_2_. Soft tissues were removed from the teeth and jaws, carefully avoiding any contact between instruments and the M_2_ crowns, and specimens were further cleaned by boiling in individual beakers of water for 2 minutes. Prior to analysis, tooth surfaces were carefully cleaned with acetone applied with a soft synthetic brush. In a few cases the first attempts at data acquisition from the M_2_ facets revealed the presence of small crystals precipitated over the functional surface. These specimens were returned for a short period to the solution in which they had been stored, were gently brushed with de-ionized water on removal, and air-dried before examination.

| **Species** | **Specimen no.** | **IFM sample no.** | **PCA plot no.** | **Source and details** |
| --- | --- | --- | --- | --- |
| *Rhinolophus ferrumequinum* | LEIUG 123048 | 11002 | 1 | UOB; Brockley, N Somerset (07.04.87), male |
| *Rhinolophus ferrumequinum* | LEIUG 123049 | 11014 | 2 | UOB; (10.06.86), female |
| *Rhinolophus ferrumequinum* | LEIUG 123050 | 11019 | 3 | UOB; Brockley, N Somerset (01.08.88), female |
| *Rhinolophus ferrumequinum* | LEIUG 123051 | 11213 | 4 | UOB; Brockley, N Somerset (21.02.87), female |
| *Rhinolophus ferrumequinum* | LEIUG 123052 | 11224 | 5 | UOB |
| *Pipistrellus pygmaeus* | LEIUG 123053 | 11227 | 6 | UOB; Avon Gorge, Bristol (16.03.90), female |
| *Pipistrellus pygmaeus* | LEIUG 123054 | 11231 | 7 | UOB; (19.06.06), female |
| *Pipistrellus pygmaeus* | LEIUG 123055 | 11232 | 8 | UOB; Abergavenny, Monmouthshire (04.03) |
| *Pipistrellus pygmaeus* | LEIUG 123056 | 11235 | 9 | UOB |
| *Pipistrellus pygmaeus* | LEIUG 123057 | 11239 | 10 | VLA; formalin stored |
| *Pipistrellus pipistrellus* | LEIUG 123058 | 11416 | 16 | UOB; Fishponds, Bristol on 05.12.88, male |
| *Pipistrellus pipistrellus* | LEIUG 123059 | 11417 | 17 | UOB; male |
| *Pipistrellus pipistrellus* | LEIUG 123060 | 11420 | 18 | UOB; male |
| *Pipistrellus pipistrellus* | LEIUG 123061 | 11423 | 19 | UOB; Hotwells, Bristol on 15.09.87, male |
| *Pipistrellus pipistrellus* | LEIUG 123062 | 11425 | 20 | UOB; Avon Gorge, Bristol, male |
| *Plecotus auritus* | LEIUG 123063 | 11240 | 11 | VLA; formalin stored, enamel brittle |
| *Plecotus auritus* | LEIUG 123064 | 11247 | 12 | NLB; male |
| *Plecotus auritus* | LEIUG 123065 | 11248 | 13 | NLB; female |
| *Plecotus auritus* | LEIUG 123066 | 11255 | 14 | VLA; formalin stored, enamel brittle |
| *Plecotus auritus* | LEIUG 123067 | 11261 | 15 | VLA; formalin stored, enamel brittle |

Abbreviations: LEIUG, University of Leicester Geology collections; UOB, University of Bristol; VLA, Veterinary Laboratory Agencies; NLB, North Lancashire Bat Group.

Table S2. Short definitions and categorization of 3D areal surface texture parameters. For further explanation see Figs S1 and S2.

| parameter | unit | definition |  |
| --- | --- | --- | --- |
| Sq | µm | Root-Mean-Square height of surface | height |
| Sp | µm | Maximum peak height of surface | height |
| Sv | µm | Maximum valley depth of surface | height |
| Sz | µm | Maximum height of surface | height |
| Sa | µm | Average height of surface | height |
| Ssk | - | Skewness of height distribution of surface | height |
| Sku | - | Kurtosis of height distribution of surface | height |
| S5z | µm | 10 point height of surface | feature |
| Sdq | - | Root mean square gradient of the surface | hybrid |
| Sdr | % | Developed interfacial area ratio | hybrid |
| Sds | 1/mm^2^ | Density of summits. Number of summits per unit area making up the surface | hybrid |
| Ssc | 1/µm | Mean summit curvature for peak structures |  |
| Sk | µm | Core roughness depth, Height of the core material | material ratio |
| Spk | µm | Mean height of the peaks above the core material | material ratio |
| Svk | µm | Mean depth of the valleys below the core material | material ratio |
| Smr1 | % | Surface bearing area ratio (the proportion of the surface which consists of peaks above the core material) | material ratio |
| Smr2 | % | Surface bearing area ratio (the proportion of the surface which would carry the load) | material ratio |
| Vmp | µm^3^/mm^2^ | Material volume of the peaks of the surface | volume |
| Vmc | µm^3^/mm^2^ | Material volume of the core of the surface | volume |
| Vvc | µm^3^/mm^2^ | Void volume of the core of the surface | volume |
| Vvv | µm^3^/mm^2^ | Void volume of the valleys of the surface | volume |
| Sal | mm | Auto correlation length. Horizontal distance of the auto correlation function (ACF) which has the fastest decay to the value 0.2. Large value: surface dominated by low frequencies. Small value: surface dominated by high frequencies. | spatial |
| Str | - | Texture aspect ratio (values range 0-1). Ratio from the distance with the fastest to the distance with the slowest decay of the ACF to the value. 0.2-0.3: surface has a strong directional structure. > 0.5: surface has rather uniform texture. | spatial |


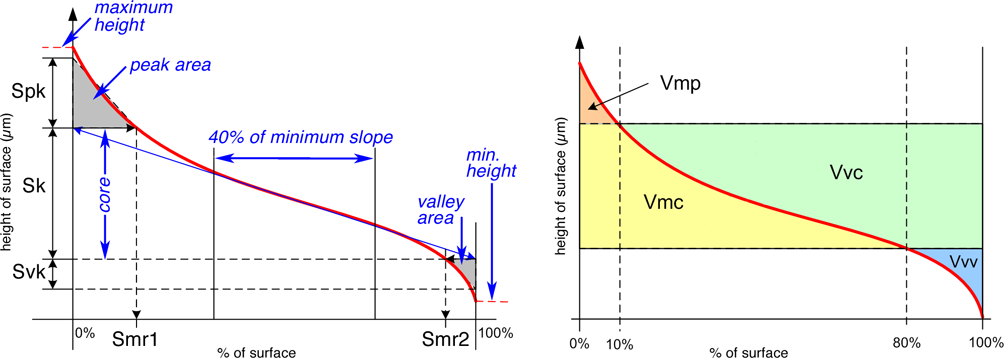


Figure S1. The Areal Material Ratio Curve (also referred to as the Bearing Area Curve, or Abbot-Firestone Curve) from which a number of height, volume, and material ratio parameters are derived. For definitions of parameters, see Table S1. The curve is a cumulative probability density function, derived from the scale limited surface by plotting the cumulative percentage of the surface against height. Core, peaks and valleys within a surface are defined on the basis of this curve, with the core equivalent to the volume that lies between the heights of the surface delimited by the extrapolated intercept of the minimum slope of the curve as shown in the figure. Modified with permission from Alicona Infinite Focus Manual.


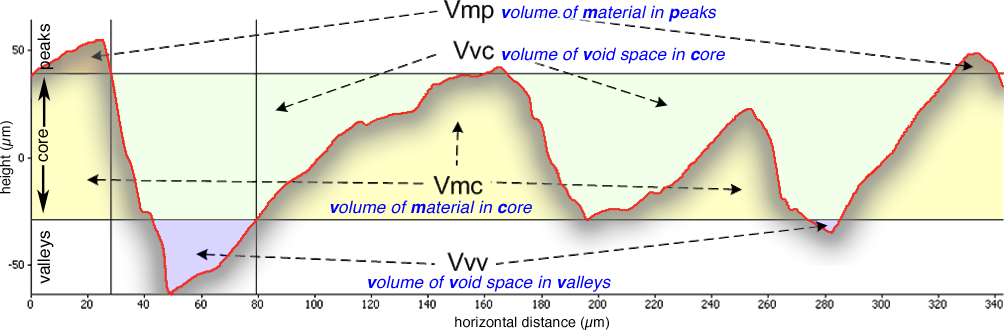


Figure S2. Cross section through a surface showing how volume parameters relate to a surface. Note that this is a 2D profile, but the parameters are volumes calculated for the whole surface. Modified with permission from Alicona Infinite Focus Manual.

Table S3. Loadings (eigenvectors) for roughness parameters onto PC axes 1 and 2 for the PCA analysis (9 parameters that differ significantly between bat species).

| Parameter | Axis 1 | Axis 2 |
| --- | --- | --- |
| Ssk | 0.447 | 0.084 |
| Str | 0.404 | 0.114 |
| Vmp (µm^3^/mm^2^) | 0.104 | 0.503 |
| Vmc (µm^3^/mm^2^) | -0.199 | 0.463 |
| Vvc (µm^3^/mm^2^) | -0.117 | 0.500 |
| Vvv (µm^3^/mm^2^) | -0.326 | 0.382 |
| Svk (µm) | -0.382 | 0.113 |
| Smr1 (%) | 0.391 | 0.245 |
| Smr2 (%) | 0.410 | 0.211 |
